# Brachial artery peak velocity variation to predict fluid responsiveness in mechanically ventilated patients

**DOI:** 10.1186/cc8027

**Published:** 2009-09-03

**Authors:** Manuel Ignacio Monge García, Anselmo Gil Cano, Juan Carlos Díaz Monrové

**Affiliations:** 1 Servicio de Cuidados Críticos y Urgencias, Unidad de Investigación Experimental, Hospital del SAS Jerez, C/Circunvalación s/n, 11407, Jerez de la Frontera, Spain

## Abstract

**Introduction:**

Although several parameters have been proposed to predict the hemodynamic response to fluid expansion in critically ill patients, most of them are invasive or require the use of special monitoring devices. The aim of this study is to determine whether noninvasive evaluation of respiratory variation of brachial artery peak velocity flow measured using Doppler ultrasound could predict fluid responsiveness in mechanically ventilated patients.

**Methods:**

We conducted a prospective clinical research in a 17-bed multidisciplinary ICU and included 38 mechanically ventilated patients for whom fluid administration was planned due to the presence of acute circulatory failure. Volume expansion (VE) was performed with 500 mL of a synthetic colloid. Patients were classified as responders if stroke volume index (SVi) increased ≥ 15% after VE. The respiratory variation in Vpeak_brach _(ΔVpeak_brach_) was calculated as the difference between maximum and minimum values of Vpeak_brach _over a single respiratory cycle, divided by the mean of the two values and expressed as a percentage. Radial arterial pressure variation (ΔPP_rad_) and stroke volume variation measured using the FloTrac/Vigileo system (ΔSV_Vigileo_), were also calculated.

**Results:**

VE increased SVi by ≥ 15% in 19 patients (responders). At baseline, ΔVpeak_brach_, ΔPP_rad _and ΔSV_Vigileo _were significantly higher in responder than nonresponder patients [14 vs 8%; 18 vs. 5%; 13 vs 8%; P < 0.0001, respectively). A ΔVpeak_brach _value >10% predicted fluid responsiveness with a sensitivity of 74% and a specificity of 95%. A ΔPP_rad _value >10% and a ΔSV_Vigileo _>11% predicted volume responsiveness with a sensitivity of 95% and 79%, and a specificity of 95% and 89%, respectively.

**Conclusions:**

Respiratory variations in brachial artery peak velocity could be a feasible tool for the noninvasive assessment of fluid responsiveness in patients with mechanical ventilatory support and acute circulatory failure.

**Trial Registration:**

**ClinicalTrials.gov ID**: NCT00890071

## Introduction

Traditional indices of cardiac preload, such as intracardiac pressures or telediastolic volumes, have been consistently surpassed by dynamic parameters to detect fluid responsiveness in critically ill patients [1,2]. The magnitude of cyclic changes in left ventricular (LV) stroke volume due to intermittent positive-pressure ventilation have been demonstrated to accurately reflect preload-dependence in mechanically ventilated patients [3]. So, the greater the respiratory changes in LV stroke volume, the greater the expected increase in stroke volume after fluid administration.

By increasing intrathoracic pressure and lung volume, mechanical insufflation raises both pleural and transpulmonary pressure, decreasing the pressure gradient for venous return and increasing right ventricular (RV) afterload. According to the Frank-Starling relationship, if both ventricles remain sensitive to changes in preload, RV stroke volume, and therefore LV preload, should decrease during positive-pressure inspiration, diminishing LV stroke volume after a few beats (normally during expiration). On the otherhand, if any of the ventricles are unaffected by cyclic variations of preload, LV stroke volume should be unaltered by swings in intrathoracic pressure. Therefore, the degree of respiratory variations in LV stroke volume could be used to reveal the susceptibility of the heart to changes in preload induced by mechanical insufflation [3].

In this regard, several surrogate measurements of LV stroke volume have been proposed to determine the preload-dependence status of a patient, such as pulse pressure variation [4], stroke volume variation [5] or aortic blood flow variation [6]. However, the acquisition of these parameters usually requires an invasive catheterization or a skilled echocardiographic evaluation to obtain an accurate interpretation of data, limiting their applicability because of the need for specialized training and equipment.

Recently, Brennan and colleagues [7], using a hand-carried Doppler ultrasound at the bedside, demonstrated that respiratory variations in brachial artery peak velocity (ΔVpeak_brach_), measured by clinicians with minimal ultrasound expertise, were closely correlated with radial artery pulse pressure variations (ΔPP_rad_), a well-known parameter of fluid responsiveness. Moreover, a ΔVpeak_brach _value of 16% or more was highly predictive of a ΔPP_rad _of 13% or more (the usual ΔPP_rad _threshold value for discrimination between fluid responder and nonresponder patients), so ΔVpeak_brach _could be used as a noninvasive surrogate of LV stroke volume variation for assessing preload dependence in patients receiving controlled mechanical ventilation. However, the predictive value of this indicator was not tested performing a volume challenge and checking the effects on cardiac output (CO) or stroke volume. Thus, although promising, further studies are required before validating this parameter and recommending it for its clinical use [8].

Therefore, we designed the current study to confirm the predictive value of the ΔVpeak_brach _for predicting fluid responsiveness in mechanically ventilated patients with acute circulatory failure.

## Materials and methods

This study was approved by the Institutional Ethics Committee of the Jerez Hospital of the Andalusian Health Service and endorsed by the Scientific Committee of the Spanish Society of Intensive Care, Critical and Coronary Units. Written informed consent was obtained from each patient's next of kin.

### Patients

The inclusion criteria were patients with controlled mechanical ventilation, equipped with an indwelling radial artery catheter and for whom the decision to give fluids was taken due to the presence of one or more clinical signs of acute circulatory failure, defined as a systolic blood pressure of less than 90 mmHg (or a decrease of more than 50 mmHg in previously hypertensive patients) or the need for vasopressor drugs; the presence of oliguria (urine output <0.5 ml/kg/min for at least two hours); the presence of tachycardia; a delayed capillary refilling; or the presence of skin mottling. Contraindication for the volume administration was based on the evidence of fluid overload and/or hydrostatic pulmonary edema. Patients with unstable cardiac rhythm were also excluded.

### Arterial pulse pressure variation

Radial arterial pressure was recorded online on a laptop computer at a sampling rate of 300 Hz using proprietary data-acquisition software (S/5 Collect software, version 4.0; Datex-Ohmeda, Helsinki, Finland) for further off-line analysis (QtiPlot software, version 0.9.7.6 [9].

ΔPP_rad _was defined according to the formula:

where PP_max _and PP_min _are the maximum and minimum pulse pressures determined during a single respiratory cycle, respectively [10]. The average of three consecutive determinations was used to calculate ΔPP_rad _for statistical analysis.

### Respiratory variation in brachial artery blood velocity

The brachial artery blood velocity signal was obtained using a Doppler ultrasound scanner (Vivid 3, General Electric, Waukesha, WI, USA), equipped with a 4 to 10 MHz flat linear array transducer. With the patient in the supine position, the transducer was placed over a slightly abducted arm, opposite to the indwelling radial artery catheter and 5 to 10 cm above the antecubital fossa. After confirmed correct placement and artery pulse quality by Doppler ultrasound, the transducer was rotated to acquire the transversal image of the artery. Angle Doppler was adjusted to ensure a less than 60° angle for the accurate determination of Doppler shift and blood flow velocity. The velocity waveform was recorded from the midstream of the vessel lumen and the sample volume was adjusted to cover the center of the arterial vessel, in order to obtain a clear Doppler blood velocity trace. Brachial flow velocity was registered simultaneously to the radial arterial pressure for at least one minute.

The ΔVpeak_brach _was calculated on-line using built-in software as:

where Vpeak_max _and Vpeak_min _are the maximum and the minimum peak systolic velocities during a respiratory cycle, respectively. The mean values of the three consecutive determinations were used for statistical analysis.

The intraobserver reproducibility was determined for ΔVpeak_brach _measurements using Bland-Altman test analysis in all target patients over a one-minute period, and described as mean bias ± limits of agreements.

### Cardiac output and stroke volume variation measurements

A FloTrac sensor (Edwards Lifesciences LLC, Irvine, CA, USA) was connected to the arterial line and attached to the Vigileo monitor, software version 1.10 (Edwards Lifesciences LLC, Irvine, CA, USA). Briefly, the CO was calculated from the real-time analysis of the arterial waveform over a period of 20 seconds at a sample rate of 100 Hz without prior external calibration, using a proprietary algorithm based on the relation between the arterial pulse pressure and stroke volume. Arterial compliance and vascular resistance contribution was estimated every minute based on individual patient demographic data (age, gender, body weight and height) and the arterial waveform analysis, respectively. Stroke volume variation (ΔSV_Vigileo_) was assessed by the system as follows:

A time interval of 20 seconds was used by the algorithm to calculate SV_mean _and ΔSV_Vigileo _[11].

After zeroing the system against atmosphere, the arterial waveform signal fidelity was checked using the square wave test and hemodynamic measurements were initiated. CO, stroke volume and ΔSV_Vigileo _values were obtained by an independent physician and averaged as the mean of three consecutive measurements. The Doppler operator was unaware of the Vigileo monitor measurements.

### Study protocol

All the patients were ventilated in controlled-volume mode (Puritan Bennett 840 ventilator, Tyco, Mansfield, MA, USA) and temporally paralyzed (vecuronium bromide 0,1 mg/Kg) if spontaneous inspiratory efforts were detected on the airway pressure curve displayed on the respiratory monitor. Supportive therapies, ventilatory settings and vasopressor therapy were kept unchanged throughout the study time. A first set of hemodynamic measurements was obtained at baseline and after volume expansion (VE), consisting of 500 ml of synthetic colloid (Voluven^®^, hydroxyethylstarch 6%; Fresenius, Bad Homburg, Germany) infused over 30 minutes.

### Statistical analysis

Non-parametric tests were applied as data were not normally distributed. Results are expressed as median and interquartile range (25^th ^to 75^th ^percentiles). Patients were classified according to stroke volume index (SVi) increase after VE in responders (≥15%) and nonresponders (<15%), respectively [10]. The effects of VE on hemodynamic parameters were assessed using the Wilcoxon rank sum test. Differences between responder and nonresponder patients were established by the Mann-Whitney *U *test. The rate of vasopressor treatment was compared between responder and nonresponder patients using the chi-squared test. The relations between variables were analyzed using a linear regression method. The area under the receiver operating characteristic (ROC) curves for ΔVpeak_brach_, ΔPP_rad_, ΔSV_Vigileo _and central venous pressure (CVP) according to fluid expansion response were calculated and compared using the Hanley-McNeil test. ROC curves are presented as area ± standard error (95% confidence interval (CI)). A *P *value less than 0.05 was considered statistically significant. Statistical analyses were performed using MedCalc for Windows, version 10.3.4.0 (MedCalc Software, Mariakerke, Belgium).

## Results

Thirty-eight patients were included in the study, 19 of them with an increased SVi of 15% or higher (responders). The main characteristics of the studied population are summarized in Table 1. The vasoactive rate was not different between responders and nonresponders. Neither tidal volume nor positive end-expiratory pressure (PEEP) was significantly different between both groups. Volume expansion was performed according to the presence of hypotension (n = 19; 50%), oliguria (n = 29; 76%), tachycardia (n = 18; 47%), delayed capillary refilling (n = 7; 18%) and mottled skin (n = 2; 5%). The intraobserver variability in ΔVpeak_brach _measurement was -1 ± 6.68.

**Table 1:** Characteristics and demographics data of t population (n = 38)

*Age (years)*	54.5 (45 to 69)
*Gender (M/F)*	19/19
*Body surface area (m*^2^)	1.90 (1.73 to 2.03)
*Death, n (%)*	11 (29%)
*ICU stay before inclusion (days)*	1 (1 to 2)
*Ventilator settings*	
*Tidal volume, mL/Kg ideal body weight*	9 (8 to 10)
*Respiratory rate, breaths/min*	20 (18 to 20)
*Total PEEP, cm H*_2_*O*	6 (4 to 6)
*FiO*_2_, %	63 (50 to 80)
*SaO*_2_, %	98.5 (95 to 99)
*Norepinephrine, n; dose (μ/kg/min)*	17; 0.52 (0.38 to 0.9)
*Dobutamine, n; dose (μg/kg/min)*	4; 7.11 (4.89 to 11.04)
*Sepsis, n (%)*	
*Abdominal*	11 (29%)
*Pulmonary*	3 (8%)
*Neurological*	3 (8%)
*Urological*	2 (5%)
*Skin and soft tissues*	1 (3%)

Values are expressed as absolute numbers or median with interquartile range (25^th ^to 75^th ^percentiles). F: female; FiO: inspired oxygen fraction; ICU: intensive care unit; M: male; PEEP: positive end-expiratory pressure; SaO_2_ : arterial oxygen saturation.

### Hemodynamic response to volume expansion

Hemodynamic parameters before and after VE are displayed in Table 2. In the whole population, VE induced a significant percentage gain in mean arterial pressure of 9.1% (3.3 to 19%), cardiac index by 10% (2.1 to 20.1%), SVi by 29% (20.4 to 37.5%) and CVP by 60% (28.5 to 72%).

**Table 2:** Effects of volume expansion in hemodynamic parameters

	Pre VE	Post VE
**CI, *L/min/m***^2^		
*Responders*	2.85 (2.64 to 3.34)	3.74 (2.93 to 4.14)**
*Nonresponders*	2.76 (2.34 to 4.54)	2.76 (2.53 to 4.57)*
**HR, *beats/min***		
*Responders*	102 (90 to 118)†	91 (82 to 109)*
*Nonresponders*	75 (65 to 101)	77 (68 to 100)
**SVi, *mL/m***^2^		
*Responders*	30.4 (23.9 to 39)†	37.9 (32.1 to 50.6) ***
*Nonresponders*	37.8 (30.7 to 47.1)	36.9 (32 to 49)
**MAP, *mmHg***		
*Responders*	72 (62 to 78)	81 (65 to 89)**
*Nonresponders*	78 (69 to 86)	91 (76 to 100)**
**SAP, *mmHg***		
*Responders*	102 (80 to 115)†	120 (96 to 139)**
*Nonresponders*	126 (106 to 141)	141 (115 to 158)***
**DAP, *mmHg***		
*Responders*	55 (48 to 59)	58 (50 to 64)*
*Nonresponders*	52 (45 to 63)	60 (51 to 73)*
**TSVRi, *dyn· s· cm*^-5^·*m*^2^**		
*Responders*	1768 (1478 to 2066)	1662 (1333 to 1744)*
*Nonresponders*	1959 (1258 to 2271)	1930 (1236 to 2582)
**CVP, *mmHg***		
*Responders*	7 (4 to 10)	10 (7 to 12) † ***
*Nonresponders*	8 (6 to 11)	13 (11 to 16)***

Data are expressed as median with interquartile range (25^th ^to 75^th ^percentiles). * *P *< 0.05, ** *P *< 0.001, *** *P *< 0.0001 post VE vs pre VE; † *P *< 0.05 responders vs nonresponders.

CI: cardiac index; CVP: central venous pressure; DAP: diastolic arterial pressure; HR: heart rate; MAP: mean arterial pressure; SAP: systolic arterial pressure; Svi: stroke volume index; TSVRi: total systemic vascular resistance index; VE: volume expansion.

### Effects of VE on dynamic parameters of preload

**Figure 1:**
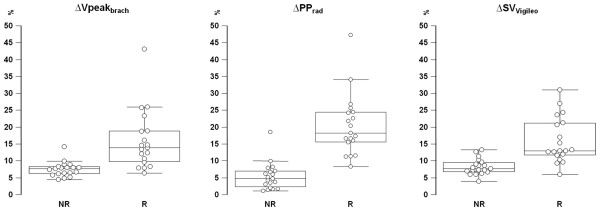
Comparison of different dynamic indices of preload. Box-and-whisker plots and individual values (*open circles*) of respiratory variations of brachial peak velocity (ΔVpeak_brach_), radial arterial pulse pressure variation (ΔPP_rad_) and stroke volume variation measured using the FloTrac/Vigileo monitoring system (ΔSV_Vigileo_) before volume expansion (VE), in responder (R, stroke volume index (SVi) ≥15% after VE) and nonresponder (NR, SVi <15% after VE) patients. The central box represents the values from the lower to upper quartile (25^th ^to 75^th ^percentile). The middle line represents the median. A line extends from the minimum to the maximum value.

**Figure 2:**
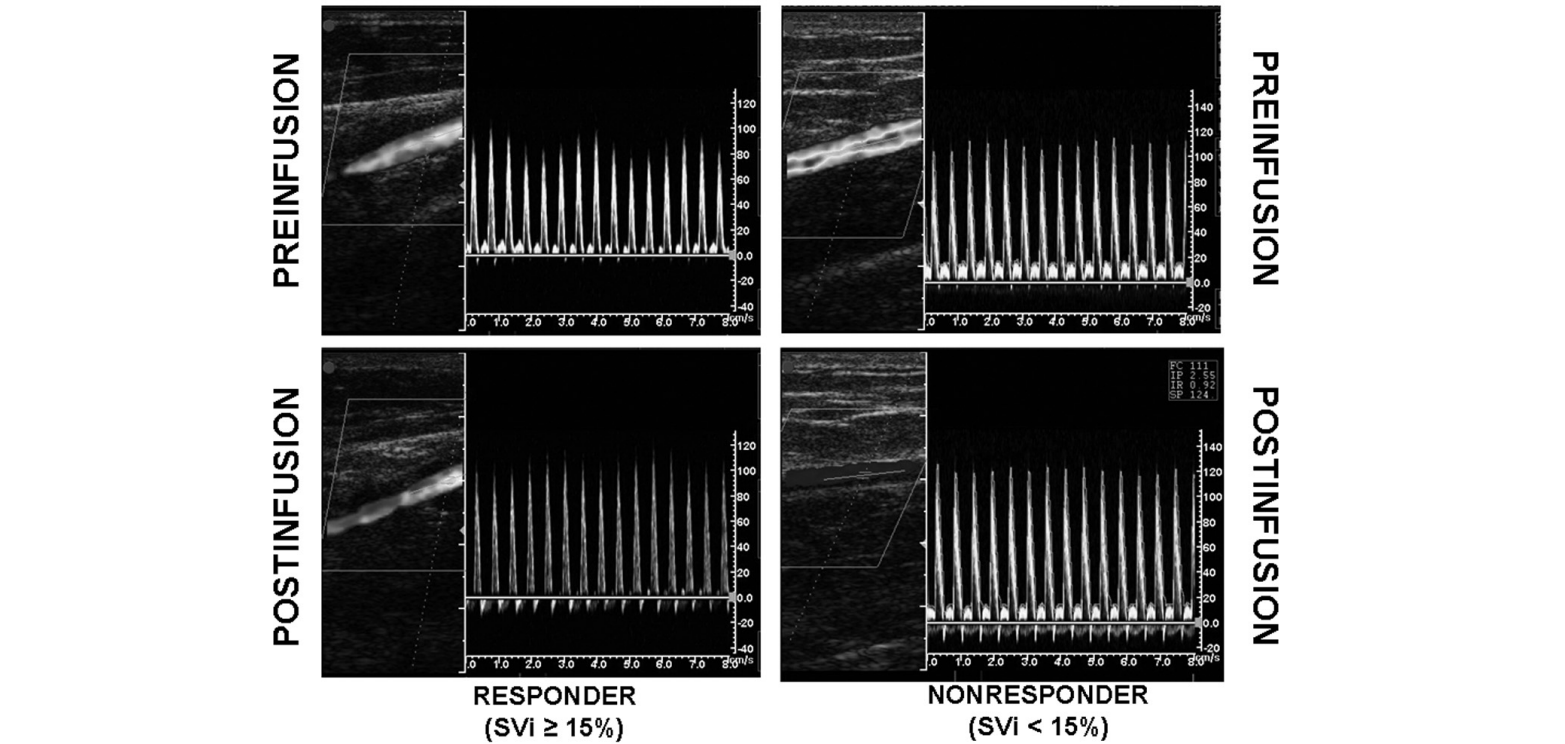
Illustrative example of Doppler evaluation of brachial artery peak velocity variation in a responder patient and nonresponder patient. In the responder patient (left), volume expansion (VE) induced a decrease of brachial artery peak velocity variation (ΔVpeak_brach_) by 15% (from 23% at baseline to 8% after VE) and an increase of stroke volume index and cardiac index by 27% and 12%, respectively. Radial pulse pressure variation (ΔPP_rad_) and stroke volume variation (ΔSV_Vigileo_) also significantly decreased in the same patient (from 23% to 4%, and from 24% to 11%, respectively). In nonresponder patients (right), VE did not induce any significant change in ΔVpeak_brach _(from 9% to 9% after VE), ΔPP_rad _(from 10% to 8%) or ΔSV_Vigileo _(from 13% to 12%). Neither cardiac index nor stroke volume index increased significantly after VE (6% and 8%, respectively). SVi = stroke volume index.

The effects of VE on dynamic parameters of preload are summarized in Table 3. Individual values are displayed in Figure 1. At baseline, dynamic parameters did not differ between patients treated with norepinephrine and without vasopressor support. Volume loading was associated with a significant decrease in ΔVpeak_brach _(3%, 1 to 6; *P *< 0.0001), ΔPP_rad _(4%, 2 to 11; *P *< 0.0001) and ΔSV_Vigileo _(3%, 1 to 6; *P *< 0.0001) in both groups. An example of effects of VE in ΔVpeak_brach _in one responder patient and other nonresponder is shown in Figure 2.

**Table 3:** Effects of volume expansion in dynamic parameters of preload (n = 38)

	Pre VE	Post VE
**ΔPP_rad_, %**		
*Responders*	18.29 (15.66 to 24.42)††	8.58 (4.59 to 12.05) † ***
*Nonresponders*	4.74 (2.32 to 6.95)	3.15 (0.96 to 5.24)*
**ΔVpeak_brach_, %**		
*Responders*	13.94 (9.88 to 18.83)††	6.95 (5.29 to 9.38)***
*Nonresponders*	7.76 (6.24 to 8.29)	5.52 (4.38 to 7.66)*
**ΔSV_Vigileo_, %**		
*Responders*	13 (11.75 to 21.17)††	9.33 (7.08 to 11.50) †† ***
*Nonresponders*	7.67 (6.75 to 9.5)	5.67 (4.67 to 6.59)**

Data are expressed as median with interquartile range (25^th ^to 75^th ^percentiles). * *P *< 0.05, ** *P *< 0.001, *** *P *< 0.0001 post VE vs pre VE. † *P *< 0.001, †† *P *= 0.0001 responders vs nonresponders.

ΔPP_rad _:radial artery pulse pressure variation; ΔSV_Vigileo _:stroke volume variation measured with FloTrac/Vigileo^® ^monitoring system; ΔVpeak_brach _:brachial artery peak velocity variation; VE: volume expansion.

### Dynamic parameters to quantify the hemodynamic effects of VE

At baseline, both ΔVpeak_brach _and ΔPP_rad _were positively correlated with VE-induced change in SVi (r^2 ^= 0.56 and r^2 ^= 0.71; *P *< 0.0001, respectively). ΔSV_Vigileo _was also correlated, although less strongly (r^2 ^= 0.32; *P *< 0.001). Therefore, the greater the respiratory variation in brachial artery peak velocity, arterial pulse pressure or stroke volume, the greater the expected SVi increase after fluid administration. The VE-induced change in ΔVpeak_brach _and ΔPP_rad _were correlated with VE change in SVi (r^2 ^= 0.58 and r^2 ^= 0.56; *P *< 0.0001, respectively), although weakly for ΔSV_Vigileo _(r^2 ^= 0.12, *P *< 0.05). So a decrease in ΔVpeak_brach _value after VE could be used to indicate a successful increase in stroke volume by fluid administration.

### Relationship between dynamic parameters of preload

Before volume administration ΔVpeak_brach _correlated with ΔPP_rad _(r^2 ^= 0.82; *P *< 0.0001) and ΔSV_Vigileo _(r^2 ^= 0.47; *P *< 0.0001). At baseline, ΔPP_rad _also correlated with ΔSV_Vigileo _(r^2 ^= 0.59; *P *< 0.0001). The VE-induced decreases in ΔVpeak_brach_, ΔPP_rad_, Vpeak_brach_, ΔSV_Vigileo_, ΔPP_rad _and ΔSV_Vigileo _were also significantly correlated (r^2 ^= 0.71; *P *< 0.0001, r^2 ^= 0.26; *P *< 0.01 and r^2 ^= 0.39; *P *< 0.0001; respectively).

### Prediction of fluid responsiveness

**Figure 3:**
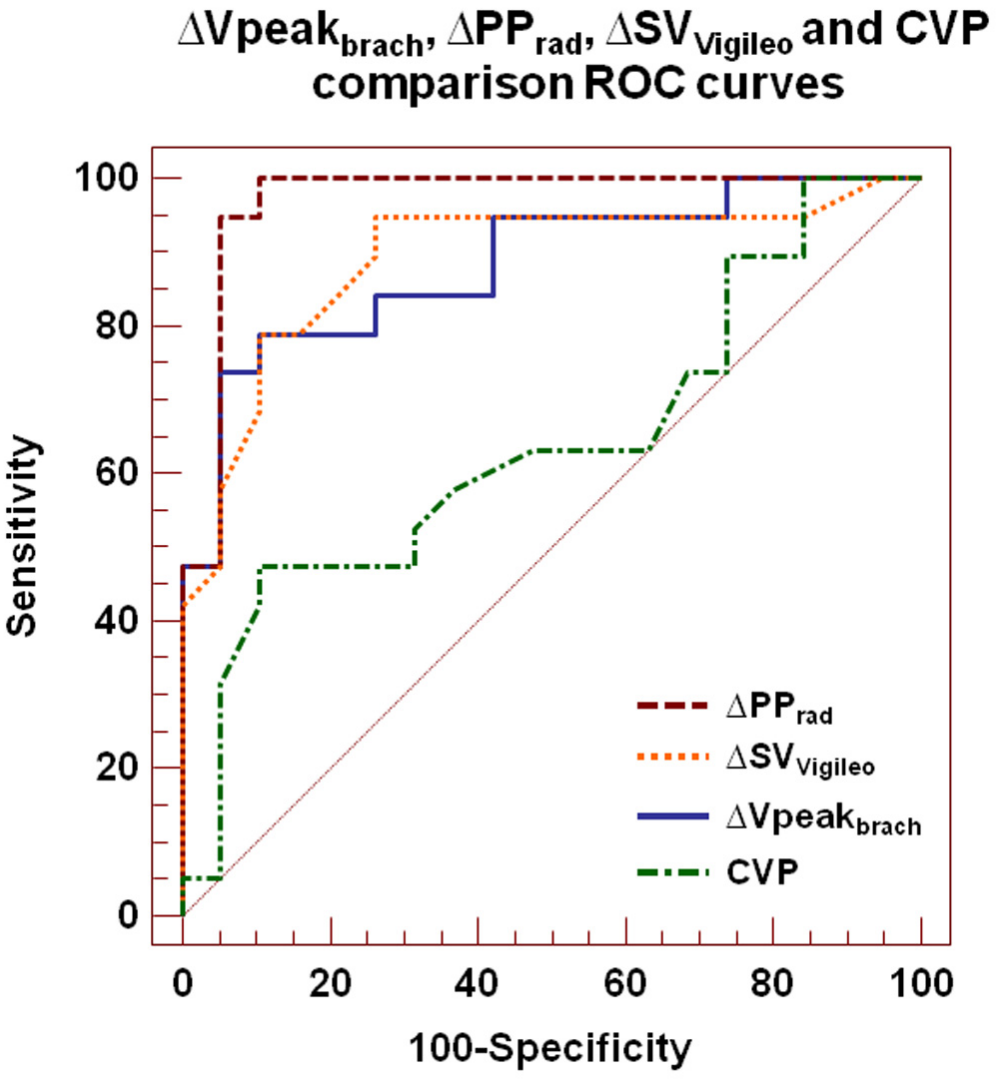
Comparison of receiver operating characteristics curves to discriminate fluid expansion responders and nonresponders. Area under the receiver operator curve (ROC) for respiratory variations of brachial peak velocity (ΔVpeak_brach_) was 0.88, for radial arterial pulse pressure variation (ΔPP_rad_) it was 0.97, for stroke volume variation measured using the FloTrac/Vigileo monitoring system (ΔSV_Vigileo_) it was 0.89 and for central venous pressure (CVP) it was 0.64.

The area under the ROC curves for baseline ΔVpeak_brach _(0.88 ± 0.06; 95% CI 0.74 to 0.96), ΔPP_rad _(0.97 ± 0.03; 95% CI 0.86 to 0.99) and ΔSV_Vigileo _(0.89 ± 0.06; 95% CI 0.75 to 0.97) was not significantly different (Figure 3). All dynamic parameters of preload were better predictors of fluid responsiveness than CVP (area under the curve: 0.64 ± 0.09; 95% CI 0.47 to 0.79). A ΔVpeak_brach _value of more than 10% predicted fluid responsiveness with a sensitivity of 74% (95% CI 49 to 91%) and a specificity of 95% (95% CI 74 to 99%), with positive and negative predictive values of 93% and 78%, respectively. A ΔPP_rad _value of more than 10% and a ΔSV_Vigileo _of more than 11% predicted volume responsiveness with a sensitivity of 95% (95% CI 74 to 99%) and 79% (95% CI 54 to 94%), and a specificity of 95% (95% CI 74 to 99%) and 89% (95% CI 67 to 97%), respectively.

## Discussion

This study demonstrates that Doppler evaluation of the ΔVpeak_brach _efficiently predicts the hemodynamic response to volume expansion in mechanically ventilated patients with acute circulatory failure.

Dynamic assessment of fluid responsiveness, unlike absolute measurements of preload, is based on the principle that challenging the cardiovascular system to a reversible and transient change on preload, the magnitude of the induced variations in stroke volume or its surrogates are proportional to the preload-dependence status of a patient [12]. Swings in intrathoracic pressure during mechanical ventilation modulate LV stroke volume by cyclically varying RV preload. As the main mechanism for reducing RV stroke volume (and hence LV filling) is impeding pressure gradient for venous return and RV preload [13], the increase in intrathoracic pressure will transiently reduce LV stroke output only if both the ventricles are operating in the steep part of the Frank-Starling curve. Therefore, phasic variations of LV stroke volume induced by positive-pressure ventilation could be used as an indicator of biventricular preload responsiveness in mechanically ventilated patients [14].

As direct measurement of LV stroke volume remains a complicated task at the bedside, different surrogate parameters have been proposed to assess the effects of mechanical ventilation in LV stroke volume for predicting volume responsiveness. In this regard respiratory variations on arterial pulse pressure [10] or the pulse contour-derived stroke volume variation [5,15] have been repeatedly demonstrated to accurately predict fluid responsiveness in different settings and clinical situations [4].

With echocardiography becoming more widely available in intensive care units and increasingly used in the management of hemodynamically unstable patients, the noninvasive assessment of preload dependence has logically aroused the interest of some authors. Feissel and colleagues [16] demonstrated that respiratory variations of aortic blood velocity, measured by transesophageal echocardiography at the level of the aortic annulus, was a more reliable parameter than a static index of preload such as LV end-diastolic area for predicting the hemodynamic response to VE in patients with septic shock. Similarly, Monnet and colleagues [6], measuring the descending aortic blood flow with an esophageal Doppler probe, reported that aortic blood flow variation and respiratory variation in aortic peak velocity were reliable indices for detecting fluid responsiveness and better predictors than the flow time corrected for heart rate, a static preload index provided by the esophageal Doppler. Moreover, in children receiving mechanical ventilation, Durand and colleagues [17], confirmed that the respiratory variation in aortic peak velocity measured by transthoracic pulsed-Doppler was superior to pulse pressure variation and systolic pressure variation for assessing cardiac preload reserve. Additionally, in two experimental studies, Slama and colleagues [18,19] analyzed the effects of controlled blood withdrawal and restitution in mechanically ventilated rabbits on the aortic velocity time integral, registered by transthoracic echocardiography, and the aortic blood flow velocity, recorded by esophageal Doppler. They observed that the prediction of hemodynamic consequences of blood depletion and restoration was highly accurate in both methods.

Recently, Brennan and colleagues [7] suggested that Doppler evaluation of respiratory variations in peak velocity of brachial artery blood flow, assessed by internal medicine residents after a brief training in brachial Doppler measurement, could be used as an easily obtainable, noninvasive surrogate of pulse pressure variation, reporting a close correlation and a high level of agreement between ΔVpeak_brach _and ΔPP_rad_. Using a ΔPP_rad _cutoff of 13% to define a positive prediction of fluid responsiveness, a ΔVpeak_brach _value of 16% or more allowed predicting with a 91% of sensitivity and 95% of specificity. Regrettably, the authors did not confirm their findings against an objective end-point of fluid responsiveness, such as changes in stroke volume or CO after a volume challenge.

Our results confirm the ability of ΔVpeak_brach _to detect preload dependence in patients receiving passive mechanical ventilation and are consistent with previously published studies by demonstrating the efficiency of dynamic parameters for predicting fluid responsiveness and its superiority over static indicators of cardiac preload. Unlike invasive indices of preload dependence, ΔVpeak_brach _measurement does not need arterial catheterization, is quickly performed at the bedside and does not require any special device or CO monitoring tool, just widely available ultrasound equipment and a minimal training in Doppler acquisition to obtain reliable measurements. Therefore, the noninvasive evaluation of mechanical ventilation over peripheral blood flow could be used as a first-line approach in the emergency department or as an initial intensive care unit assessment in hemodynamically unstable patients for whom fluid administration is considered.

When interpreting the results presented in this study some limitations must be considered. First, brachial arterial flow seems to be quite sensitive to the mechanical influence of active muscle contraction [20]. Because neuromuscular blockade was not used in all patients we cannot exclude the influence of this factor in brachial artery blood velocity measurements. However, no patient showed any spontaneous effort during the study, so the sedation level in these patients was probably deep enough to discard this possibility. Secondly, we used the FloTrac/Vigileo system for CO measurements, an uncalibrated monitoring device based on the arterial pulse contour analysis without the need for external calibration. Although the accuracy of this system has been questioned in some studies [21,22], recent papers have cited a good agreement with the thermodilution technique [23,24]. Moreover, the ability to track CO and stroke volume changes following VE seems to be comparable with the pulmonary artery catheter or the aortic Doppler-echocardiography, allowing a comparable characterization of patients according to their response to volume administration [25]. Thirdly, all surrogate parameters of LV stroke volume variations fail to predict fluid responsiveness during spontaneous ventilation or in the presence of cardiac arrhythmias [26], so our results should not be extrapolated to these clinical conditions.

## Conclusions

In conclusion, this study provides additional evidence of the utility of respiratory variations in brachial artery peak velocity induced by intermittent positive-pressure ventilation as a feasible tool for predicting fluid responsiveness, with efficiency similar to other well-known dynamic parameters of preload, in mechanically ventilated patients with acute circulatory failure.

## Key messages

• Fluid responsiveness can be reliably assessed using Doppler evaluation of ΔVpeak_brach _in mechanically ventilated patients with acute circulatory failure.

• The predictive value of ΔVpeak_brach _for assessing fluid responsiveness was similar to ΔPP_rad _and ΔSV_Vigileo_.

• A ΔVpeak_brach _value of 10% or more predicted fluid responsiveness with a 74% sensitivity and 95% specificity.

• The measurement of ΔVpeak_brach _does not need arterial catheterization, is quickly performed at the bedside and could be used as a quick first-line approach in hemodynamically unstable patients.

## Abbreviations

ΔPP_rad_: radial artery pulse pressure variation; ΔSV_Vigileo_: stroke volume variation assessed using FloTrac/Vigileo system; ΔVpeak_brach_: brachial artery peak velocity variation; ΔVpeak_max_: maximum brachial artery peak velocity during inspiration; ΔVpeak_min_: minimum brachial artery peak velocity during expiration; CI: confidence interval; CO: cardiac output; CVP: central venous pressure; LV: left ventricle; PEEP: positive end-expiratory pressure; PP_max_: maximum pulse pressure determined during a single respiratory cycle; PP_min_: minimum pulse pressure determined during a single respiratory cycle; ROC: receiver operating characteristic; RV: right ventricle; SV_max_: maximum stroke volume; SV_mean_: mean stroke volume; SV_min_: minimum stroke volume; SVi: stroke volume index; VE: volume expansion. 

## Competing interests

MIMG has received consulting fees from Edwards Lifesciences. AGC and JCDM have no conflicts of interest to disclose.

## Authors' contributions

MIMG conceived and designed the study, performed the statistical analysis, participated in the recruitment of patients and drafted the manuscript. AGC and JCDM participated in the study design, patient recruitment, measurements and data collection and helped draft the manuscript. All the authors read and approved the final manuscript.
